# Salvage Right Ventricular Outflow Tract Reconstruction for Pulmonary Embolization with Pulmonary Artery Sarcoma

**DOI:** 10.70352/scrj.cr.24-0068

**Published:** 2025-03-26

**Authors:** Tsubasa Nagai, Yusuke Misumi, Daisuke Yoshioka, Masashi Kawamura, Takuji Kawamura, Ryohei Matsuura, Ai Kawamura, Masaki Taira, Kazuo Shimamura, Daisuke Sakai, Taro Sato, Shigeru Miyagawa

**Affiliations:** 1 Department of Cardiovascular Surgery, Osaka University Graduate School of Medicine, Suita, Osaka, Japan; 2 Department of Gastroenterological Surgery, Osaka University Graduate School of Medicine, Suita, Osaka, Japan

**Keywords:** pulmonary artery intimal sarcoma, palliative surgery, right ventricular outflow tract reconstruction

## Abstract

**INTRODUCTION:**

Pulmonary artery intimal sarcoma (PAIS) is extremely rare and highly malignant. Although improved outcomes have been reported after complete surgical resection and chemotherapy, limited information is available regarding the indications, procedures, and prognosis of palliative surgery for PAIS. This report describes a successful salvage surgical case for rapid hemodynamic deterioration due to PAIS obstructing the pulmonary artery trunk.

**CASE PRESENTATION:**

A 64-year-old woman, complaining of dyspnea for a month, was referred for a pulmonary artery tumor. Imaging studies confirmed an intraluminal tumor that obstructs the pulmonary artery trunk and extends to the right ventricular wall and interventricular septum, suspecting a malignancy. During preoperative workups, she developed acute hemodynamic and respiratory deterioration due to pulmonary embolization, so emergency surgery was planned on a salvage basis. The tumor originated from the pulmonary artery intima just distal to the pulmonary valve, obstructed the pulmonary artery trunk, and extensively involved the left main coronary artery and the interventricular septum, where complete resection of the tumor was not achieved. Reconstruction of the pulmonary valve, the right ventricular outflow tract (RVOT), and bilateral pulmonary arteries were performed using a composite of a prosthetic valve and vascular grafts. The patient’s postoperative course was uneventful, and she was discharged home asymptomatic. Pathological diagnosis of the operative specimen confirmed pulmonary intimal sarcoma. After 4 months of postoperative chemotherapy, tumor progression was confirmed. The patient passed away at home 8 months after surgery.

**CONCLUSION:**

We reported a case of PAIS presenting with RVOT obstruction and rapid hemodynamic and respiratory deterioration, who underwent succeeding emergent surgery and was discharged home asymptomatic. Palliative RVOT reconstruction can be a useful surgical option for PAIS accompanying pulmonary embolization on a salvage basis.

## Abbreviations

CT
: computed tomography
FDG-PET
: fluorine-18 fluorodeoxyglucose positron emission tomography
PAIS
: pulmonary artery intimal sarcoma
RVOT
: right ventricular outflow tract

## INTRODUCTION

Pulmonary artery intimal sarcoma (PAIS) is a rare malignant disease, with an estimated incidence ranging from 0.001% to 0.03%.^1–3)^ Contrary to improved survival rates that have been reported after complete resection of the tumor and concurrent use of chemoradiotherapy, clinical outcomes are extremely poor in cases where complete extirpation is not achieved, for whom the surgical indication remains to be determined.^3–6)^ In some clinical scenarios, PAIS presents as a massive pulmonary embolism that requires emergent surgery on a salvage basis.^7–11)^ However, there are scarce reports available regarding the detailed periprocedural clinical outcomes of palliative salvage surgery for PAIS presenting as pulmonary embolization. Here, we report a case of PAIS presenting with right ventricular outflow tract (RVOT) obstruction, who underwent succeeding emergent palliative RVOT reconstruction and was discharged home asymptomatic.

## CASE PRESENTATION

**Fig. 1:**
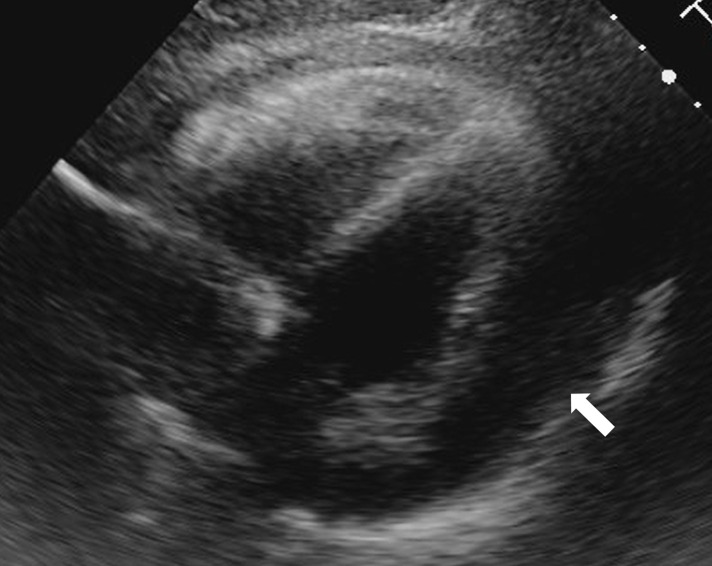
Preoperative transthoracic echocardiography showed a massive pericardial effusion (arrow).

**Fig. 2:**
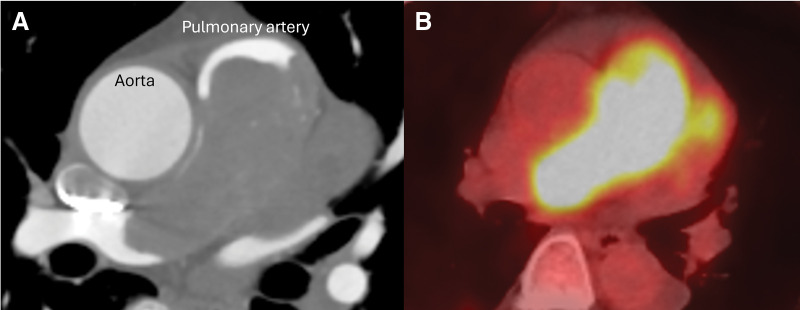
Preoperative **(A)** contrast-enhanced computed tomography showed a tumor embolizing the pulmonary artery trunk, involving the right ventricular wall and interventricular septum, and **(B)** fluorine-18 fluorodeoxyglucose positron emission tomography showed glucose activity at the pulmonary artery mass with a maximum standardized uptake value of 13.0, which is suggestive of a malignant cardiac tumor.

A 64-year-old woman was referred to our center for a suspected pulmonary artery tumor. The patient initially presented with dyspnea for a month. Her past medical history was unremarkable. On admission, the patient presented with dyspnea at rest and required oxygen therapy. Transthoracic echocardiography confirmed a large mass obstructing the pulmonary artery trunk, associated with severe pulmonary hypertension and a tricuspid regurgitation pressure gradient of 89 mmHg, as well as significant pericardial effusion (**Fig. 1**). Contrast-enhanced computed tomography (CT) confirmed an intraluminal defect that obstructs the pulmonary artery trunk and extends to the right ventricular wall and interventricular septum. Fluorine-18 fluorodeoxyglucose positron emission tomography (FDG-PET) showed glucose activity in the intraluminal defects at the pulmonary artery with a maximum standardized uptake value of 13.0, as well as no other hyperdynamic lesion (**Fig. 2**). These findings led to the diagnosis of pulmonary artery sarcoma. During further workups, she experienced rapid deterioration and respiratory failure. Therefore, emergency surgery was planned to relieve the obstruction of the RVOT.

**Fig. 3:**
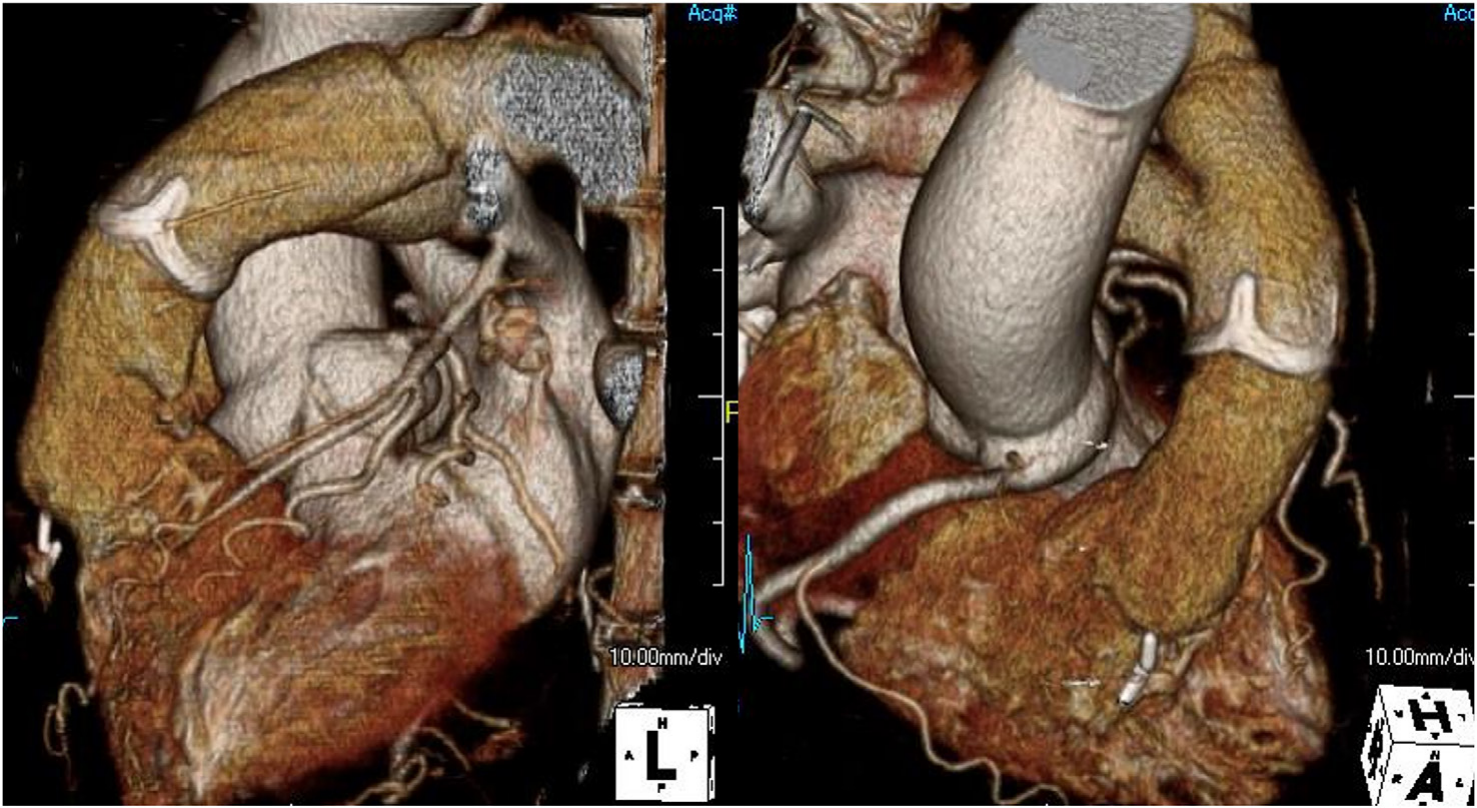
Postoperative contrast-enhanced computed tomography showed complete reconstruction of the RVOT and both pulmonary arteries using a prosthetic valve and vascular grafts. RVOT, right ventricular outflow tract

Before induction of general anesthesia, percutaneous cardiopulmonary support was introduced to stabilize hemodynamics. The operation was performed through a median sternotomy. Rapid intraoperative pathologic examination revealed no malignancy in the pericardial fluid cytology. Cardiopulmonary bypass was established with ascending aorta and bicaval cannulation. A tumor was observed on the epicardium, located between the left atrial appendage and pulmonary artery trunk, adhering to the pericardium. A pulmonary arteriotomy was performed. The tumor occupied the entire lumen of the pulmonary artery trunk and was found to have arisen from the posterior side of the pulmonary artery trunk just above the left cusp of the pulmonary valve. The tumor was resected along with the adhering pulmonary valve, pulmonary artery trunk, and right pulmonary artery just proximal to the origin of the first branch to the right upper lobe. The tumor was found to involve the left main trunk and the muscular part of the interventricular septum. Complete resection of the tumor, including the left main coronary artery and interventricular septum, was not considered feasible due to indistinct tumor margins. A palliative approach focused on tumor debulking and prosthetic reconstruction was chosen to avoid a highly invasive procedure with extensive reconstruction, including coronary artery bypass grafting and interventricular septum reconstruction. The RVOT and pulmonary valve were reconstructed with a 24-mm prosthetic vascular graft composite with a 21-mm biological valve, without cardiac arrest. The right pulmonary artery was also reconstructed with a 16-mm prosthetic vascular graft, which was anastomosed to a 24-mm vascular graft. The proximal end of the composite graft was sutured to the interventricular septum, where there was no evident tumor invasion. The patient was uneventfully weaned from cardiopulmonary bypass and returned to the intensive care unit in hemodynamically stable condition. Postoperative CT showed complete reconstruction of the RVOT and distal pulmonary arteries (**Fig. 3**).

**Fig. 4:**
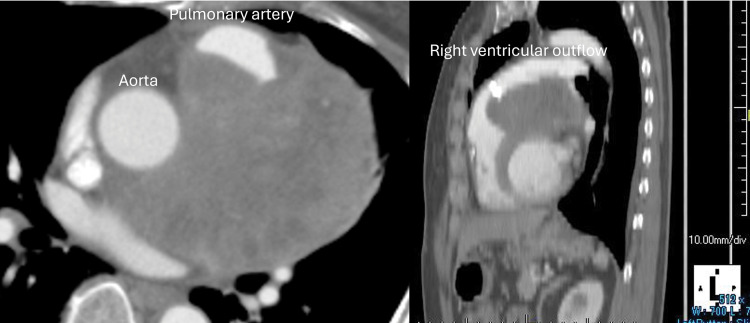
At 4 months after surgery, computed tomography showed progression of mediastinal tumor.

The postoperative course was uneventful, and the patient was discharged home on postoperative day 18. Pathological analysis of the tumor confirmed a diagnosis of malignant intimal sarcoma. Postoperative chemotherapy with gemcitabine and docetaxel was administered until the third course, when enlargement of the mediastinal tumor and metastasis to other organs were confirmed by chest X-ray and CT. Chemotherapy was discontinued at the patient’s request (**Fig. 4**). The patient expired 8 months postoperatively after 2 weeks of palliative care for dyspnea.

## DISCUSSION

PAIS is extremely rare but often associated with catastrophic clinical outcomes. While complete resection of the tumor is associated with improved survival, limited data are available regarding the clinical significance of palliative surgery aimed at salvaging hemodynamic instability or cytoreduction of the tumor.^1–3)^ We reported a successful surgical case for acute decompensated heart failure requiring temporary mechanical support due to pulmonary obstruction with PAIS.

The growing PAIS possibly obstructs the RVOT, leading to severe right ventricular dysfunction and systemic hemodynamic instability, indicating the need for temporary mechanical cardiac support or emergent surgery. Hieu et al. reported a case of PAIS obstructing the RVOT with rapid progression of respiratory failure. The patient survived an emergent pulmonary embolectomy with incomplete resection of PAIS and eventually died 1 month after surgery.^11)^ Another emergency surgical case was reported by Esaki et al. for RVOT obstruction due to malignancy. The patient underwent right ventricle and RVOT reconstruction with a procaine pericardial patch and a Dacron patch was performed. The patient was then safely discharged home but died 3 months after surgery.^7)^ In the current case, the patient developed rapid hemodynamic deterioration but was successfully bridged with temporary mechanical circulatory support to a palliative RVOT reconstruction. The patient was discharged home without symptoms and survived as long as 8 months after surgery. This case illustrates the importance of recognizing the possibility of emergent surgical indication for hemodynamic instability caused by RVOT obstruction with rapidly growing PAIS and also the potential role of salvage surgery to relieve RVOT embolization.

Another important point is the procedure for palliative surgery. Complete resection of the tumor and adjuvant chemotherapy are significantly associated with improved outcomes, though little is known regarding the relationship between the degree of tumor debulking and clinical outcome in cases with incomplete resection of the tumor. Some case series indicate months of survival after palliative surgeries. Bandyopadhyay et al. analyzed 391 cases of pulmonary artery sarcoma and reported a nearly 50% survival rate at 1 month in patients with incomplete resection of the tumor.^3)^ A report from Blackmon et al. included 65 patients with pulmonary artery sarcoma. The median survival was 11 months after tumor debulking, palliative pneumonectomy, exploration, or thromboendarterectomy.^5)^ Several case reports provide procedural details of salvage surgeries. In a case reported by Hieu et al., a salvage pulmonary endarterectomy was performed within a few weeks of symptom resolution.^11)^ In another case reported by Chang et al., complete reconstruction of the RVOT and bilateral pulmonary arteries was performed, and the residual tumor demonstrated a significant reduction in size following postoperative adjuvant chemotherapy, with the patient achieving several months of survival.^12)^ In the current case, the pulmonary valve, RVOT, and both right and left pulmonary arteries were reconstructed with a prosthetic valve and grafts, achieving 8 months of survival despite discontinuation of chemotherapy at 4 months after surgery. Our deliberate strategy, which consisted of tumor debulking and prosthetic RVOT reconstruction, carefully balanced sufficient tumor volume reduction to alleviate symptoms with minimal surgical invasiveness to achieve early recovery in a patient with a suspected poor prognosis due to high malignancy. In cases of incomplete resection, the residual tumor possibly grows to compress the reconstructed pulmonary arteries and finally leads to respiratory and/or circulatory failure. Prosthetic graft reconstruction of pulmonary arteries might be a simple and effective palliative surgical option to ensure longer survival by reconstructing a larger pulmonary artery lumen to “buy” time before the recurring tumor completely obstructs the pulmonary circulation again.

## CONCLUSION

We reported a case of PAIS presenting with RVOT obstruction and rapid hemodynamic deterioration, who underwent succeeding emergent surgery and was discharged home asymptomatic. Palliative RVOT reconstruction can be a useful option for PAIS accompanying pulmonary embolization on a salvage basis.

## DECLARATIONS

### Funding

None.

### Authors’ contributions

TN, YM, DY, DS, TS, and SM analyzed and interpreted the patient data and wrote the paper.

TN, YM, DS, and TS were involved in data collection.

DY, MK, TK, RM, AK, MT, KS, and SM were involved in reviewing the manuscript.

All authors have read and approved the final version of the manuscript.

### Availability of data and materials

The datasets used in this study are available from the corresponding author upon reasonable request.

### Ethics approval and consent to participate

The study protocol was approved by the Institutional Review Board of Osaka University (Reference no. 08218-6). Since individual patients were not identified in this study, the requirement for informed consent was waived. All baseline and clinical characteristics were obtained from the medical record of patients.

### Consent for publication

The patient provided written informed consent for the publication of this paper.

### Competing interests

The authors declare that they have no competing interests.
